# Supplementary material for: Spiroplasma Infection among Ixodid Ticks Exhibits Species Dependence and Suggests a Vertical Pattern of Transmission
Source: Microorganisms. 2021 Feb 8;9(2):333. doi: 10.3390/microorganisms9020333 (PMC7915285; doi:10.3390/microorganisms9020333)
Supplement: Supplementary file 1 [file microorganisms-09-00333-s001.pdf]

Supplementary Data

Table S1. Multicollinearity analysis of predictive variables. Variable generalized variance inflation factor (GVIF), degree of freedom (DF), $GVIF^{1/2Df}$, and variance inflation factor (VIF) are shown.

**Variable**	**GVIF**	**Df**	**$GVIF^{1/2Df}$**	**VIF**
Species	617.75	23	1.15	1.32
District	5596.75	19	1.25	1.57
Season	21.615	2	2.16	4.65
Sex	2.459	5	1.09	1.20
Year	4.80	1	2.19	4.90

Table S2. A summary for the Linear mixed models (LMM) testing the probability of infection of *Spiroplasma* allele G1.

Model: G1 ~ Species + (1 | District)								
**Responsive variable**	**Random variable**	**Fixed variable (Species)**	**Estimate**	**Std. Error**	**df**	**t value**	**Pr(>|t|)**	**Sig.**
G1	District	(Intercept)	0	0.098	367.619	0	0.99991786	
		A. testudinarium	-0.001	0.104	403.269	-0.008	0.99387137	
		D. taiwanensis	0.004	0.112	450.32	0.037	0.97015435	
		H. concinna	-0.115	0.15	586.206	-0.768	0.44255937	
		H. cornigera	-0.002	0.187	654.736	-0.008	0.99357432	
		H. flava	0.009	0.101	377.066	0.087	0.93051681	
		H. formosensis	0	0.1	402.372	-0.005	0.99640844	
		H. hystricis	0	0.101	412.673	0.004	0.99693234	
		H. japonica	-0.071	0.106	375.576	-0.668	0.50454568	
		H. kitaokai	0.003	0.101	384.617	0.025	0.9800855	
		H. longicornis	-0.003	0.1	358.143	-0.03	0.97577986	
		H. megaspinosa	-0.009	0.101	375.833	-0.093	0.92633048	
		H. yeni	-0.001	0.189	578.471	-0.003	0.99748824	
		I. monospinosus	0.044	0.107	350.094	0.414	0.67909879	
		I. nipponensis	0.016	0.134	571.389	0.122	0.90290537	
		I. ovatus	0.269	0.102	329.974	2.641	0.00866639	**
		I. pavlovsky	-0.115	0.106	353.225	-1.089	0.27679327	
		I. persulcatus	-0.054	0.103	341.04	-0.528	0.59808309	
		I. tanuki	0.041	0.187	644.472	0.221	0.82501086	
		I. turdus	0	0.118	499.332	0.004	0.99697248	

Sig. codes: 0 ‘***’ 0.001 ‘**’ 0.01 ‘*’ 0.05 ‘.’ 0.1

Table S3. A summary for the Linear mixed models (LMM) testing the probability of infection of *Spiroplasma* allele G9.

Model: G9 ~ Species + (1 | District)								
**Responsive variable**	**Random variable**	**Fixed variable (Species)**	**Estimate**	**Std. Error**	**df**	**t value**	**Pr(>|t|)**	**Sig.**
G9	District	(Intercept)	0.02	0.11	314.88	0.19	0.85	
		A. testudinarium	0.01	0.12	414.65	0.08	0.94	
		D. taiwanensis	0.01	0.13	453.42	0.09	0.93	
		H. concinna	0.08	0.16	584.90	0.46	0.65	
		H. cornigera	-0.06	0.20	660.99	-0.32	0.75	
		H. flava	-0.06	0.12	387.75	-0.52	0.61	
		H. formosensis	0.00	0.11	419.82	0.01	0.99	
		H. hystricis	0.03	0.11	433.25	0.28	0.78	
		H. japonica	0.05	0.12	388.12	0.42	0.68	
		H. kitaokai	0.26	0.11	399.05	2.30	0.02	*
		H. longicornis	-0.04	0.12	371.53	-0.37	0.71	
		H. megaspinosa	-0.01	0.12	395.01	-0.13	0.90	
		H. yeni	-0.03	0.21	560.92	-0.13	0.90	
		I. monospinosus	0.05	0.12	373.38	0.42	0.68	
		I. nipponensis	0.03	0.15	567.66	0.23	0.82	
		I. ovatus	0.06	0.12	346.24	0.55	0.58	
		I. pavlovsky	0.08	0.12	374.19	0.62	0.54	
		I. persulcatus	0.12	0.12	354.71	1.01	0.31	
		I. tanuki	0.05	0.20	649.46	0.23	0.82	
		I. turdus	0.14	0.13	503.63	1.03	0.30	

Sig. codes: 0 ‘***’ 0.001 ‘**’ 0.01 ‘*’ 0.05 ‘.’ 0.1

Table S4. A summary for the Linear mixed models (LMM) testing the probability of infection of *Spiroplasma* allele G11.

**Model: G11 ~ Species + (1 | District)**								
**Responsive variable**	**Random variable**	**Fixed variable (Species)**	**Estimate**	**Std. Error**	**df**	**t value**	**Pr(>|t|)**	**Sig.**
G11	District	(Intercept)	0.00	0.08	338.90	-0.05	0.96394978	
		A. testudinarium	0.00	0.09	431.73	-0.02	0.98305023	
		D. taiwanensis	-0.01	0.09	469.60	-0.11	0.91489436	
		H. concinna	0.15	0.12	593.97	1.24	0.21700625	
		H. cornigera	0.00	0.15	663.42	0.00	0.99782921	
		H. flava	-0.02	0.09	405.42	-0.26	0.79882098	
		H. formosensis	0.00	0.08	436.55	-0.03	0.97368613	
		H. hystricis	0.00	0.09	449.44	-0.04	0.96559932	
		H. japonica	0.09	0.09	405.86	0.96	0.34013798	
		H. kitaokai	-0.01	0.09	416.39	-0.10	0.92404503	
		H. longicornis	0.00	0.09	389.40	0.02	0.9831398	
		H. megaspinosa	0.01	0.09	412.32	0.11	0.91248783	
		H. yeni	0.00	0.16	572.21	0.01	0.98850086	
		I. monospinosus	-0.11	0.09	390.96	-1.20	0.22907682	
		I. nipponensis	-0.04	0.11	577.94	-0.32	0.75054918	
		I. ovatus	0.25	0.09	364.23	2.80	0.00534877	*
		I. pavlovsky	0.15	0.09	391.95	1.68	0.09443026	.
		I. persulcatus	0.10	0.09	372.78	1.19	0.23525984	
		I. tanuki	-0.12	0.15	652.95	-0.78	0.4341455	
		I. turdus	-0.01	0.10	517.54	-0.05	0.95833159	

Sig. codes: 0 ‘***’ 0.001 ‘**’ 0.01 ‘*’ 0.05 ‘.’ 0.1

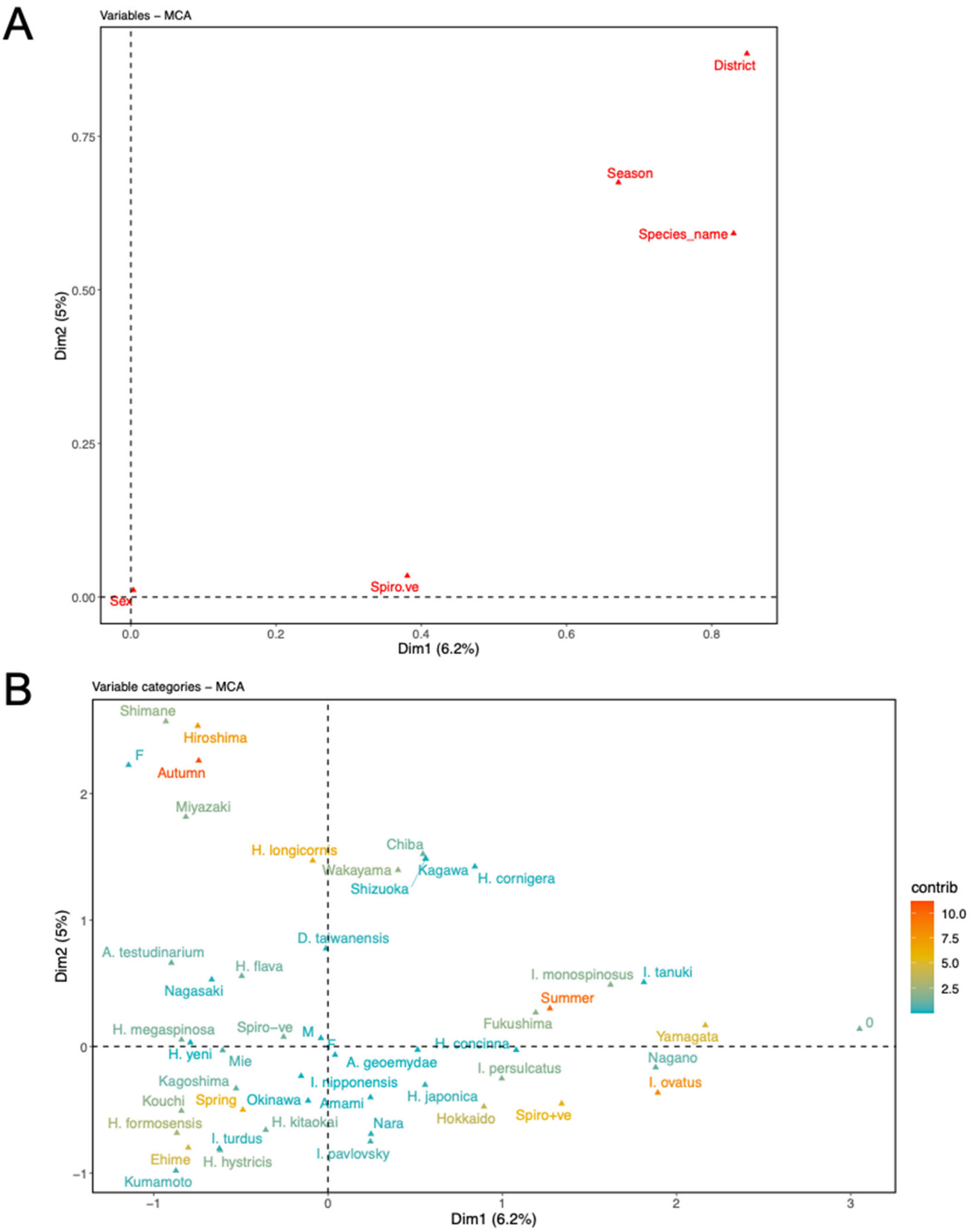

Figure S1. The results of multiple correspondence analysis. (A) Multiple correspondence analysis representing the associations between district, species, season, sex and *Spiroplasma* infection. (B) Detailed correlations between the predictors and *Spiroplasma* infection (positive and negative samples) are shown in the plot with the legend indicating the degree of variable contribution to the dimensions.

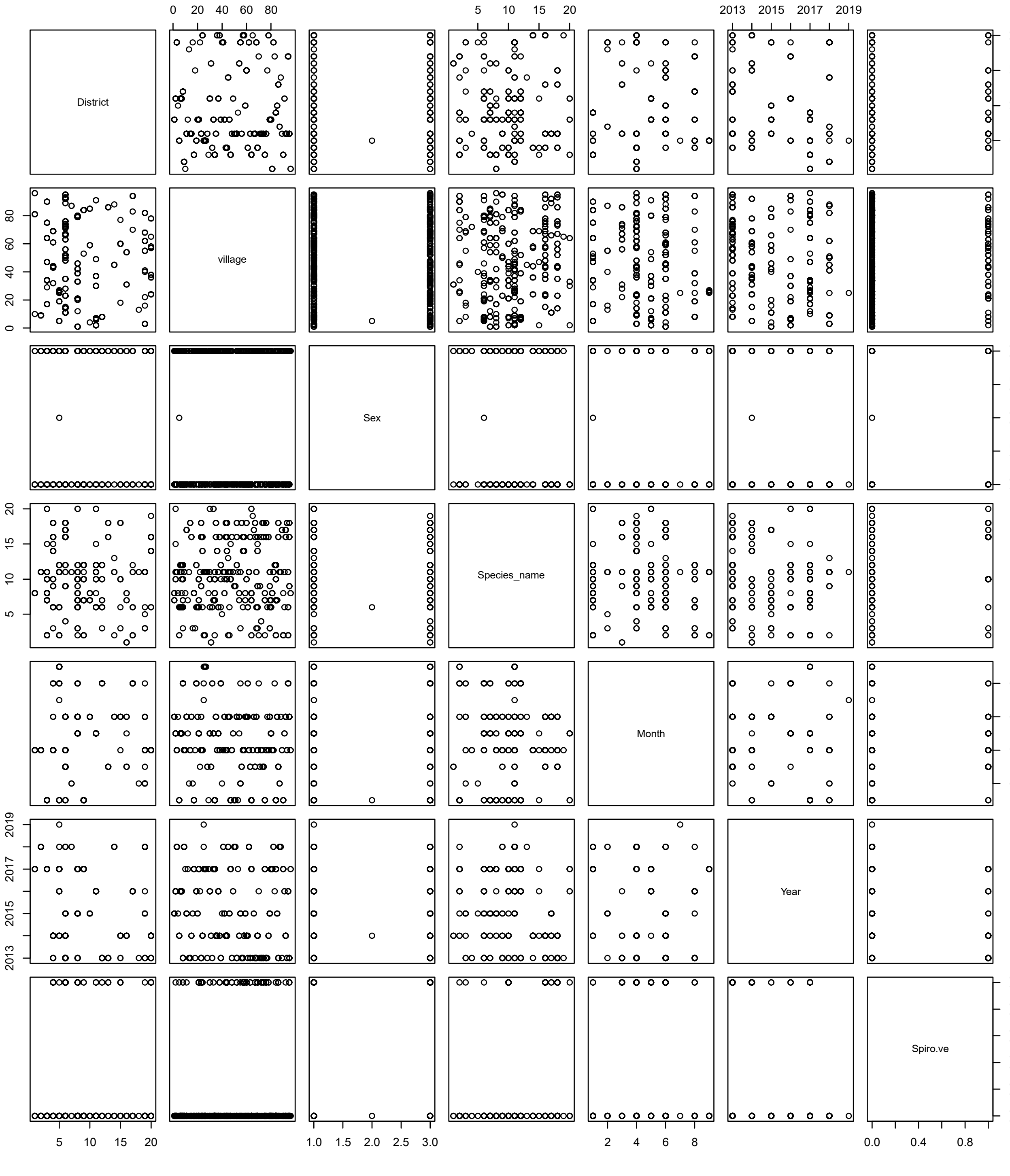

Figure S2. Multicollinearity analysis of predictive variables. Each scatter plot represents the correlation between two predictor variables.

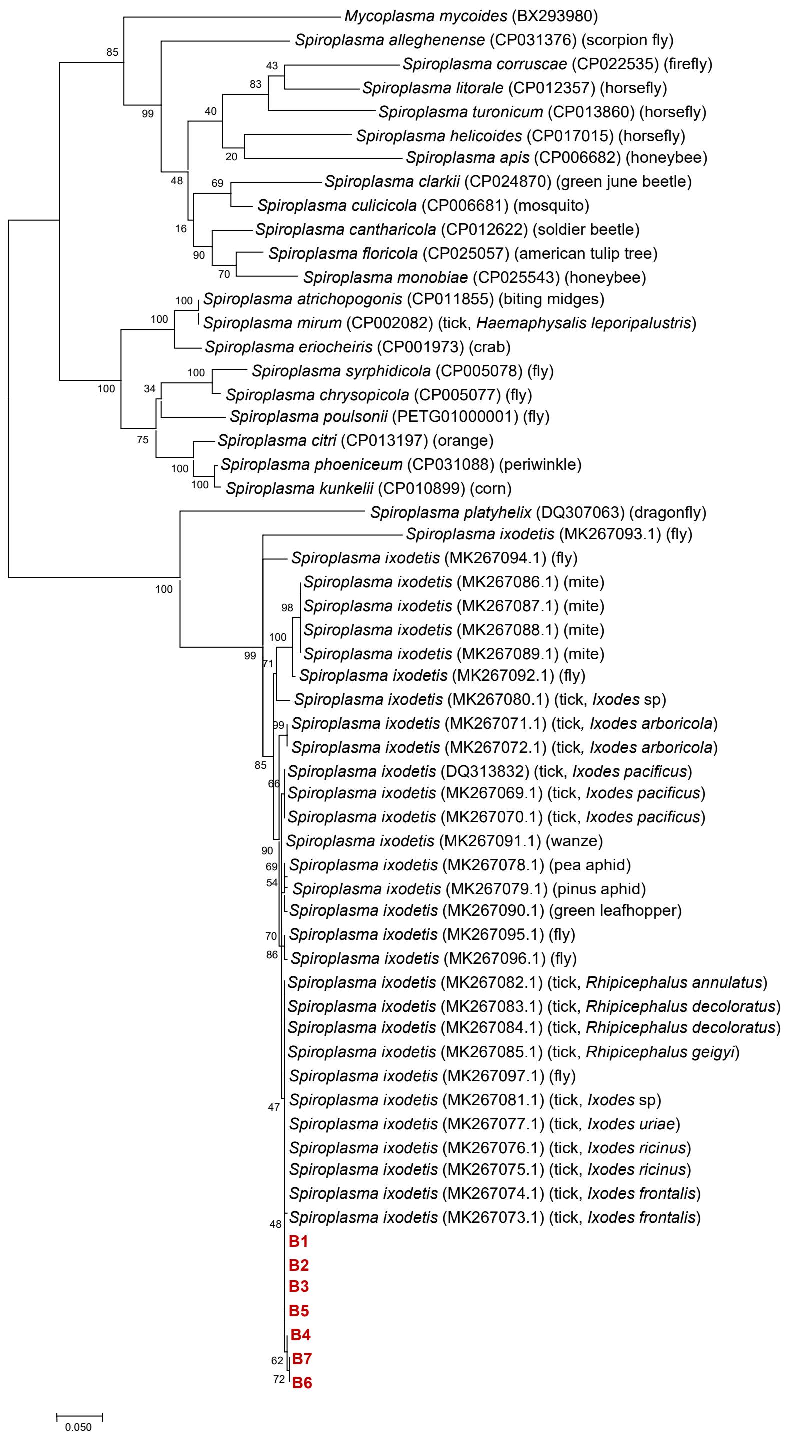

Figure S3. A phylogenetic tree based on the sequences of *rpoB* gene. The analysis was performed using a maximum-likelihood method based on the General Time Reversible model. A discrete Gamma distribution was used to model evolutionary rate differences among sites [5 categories (+ G + I, parameter = 0.5802)] with bootstrap tests of 1,000 replicates in MEGA7. The sequences obtained in this study are included with allele names provided in Table 3 and are shown in red. The sequences of other *Spiroplasma* species were retrieved from GenBank.

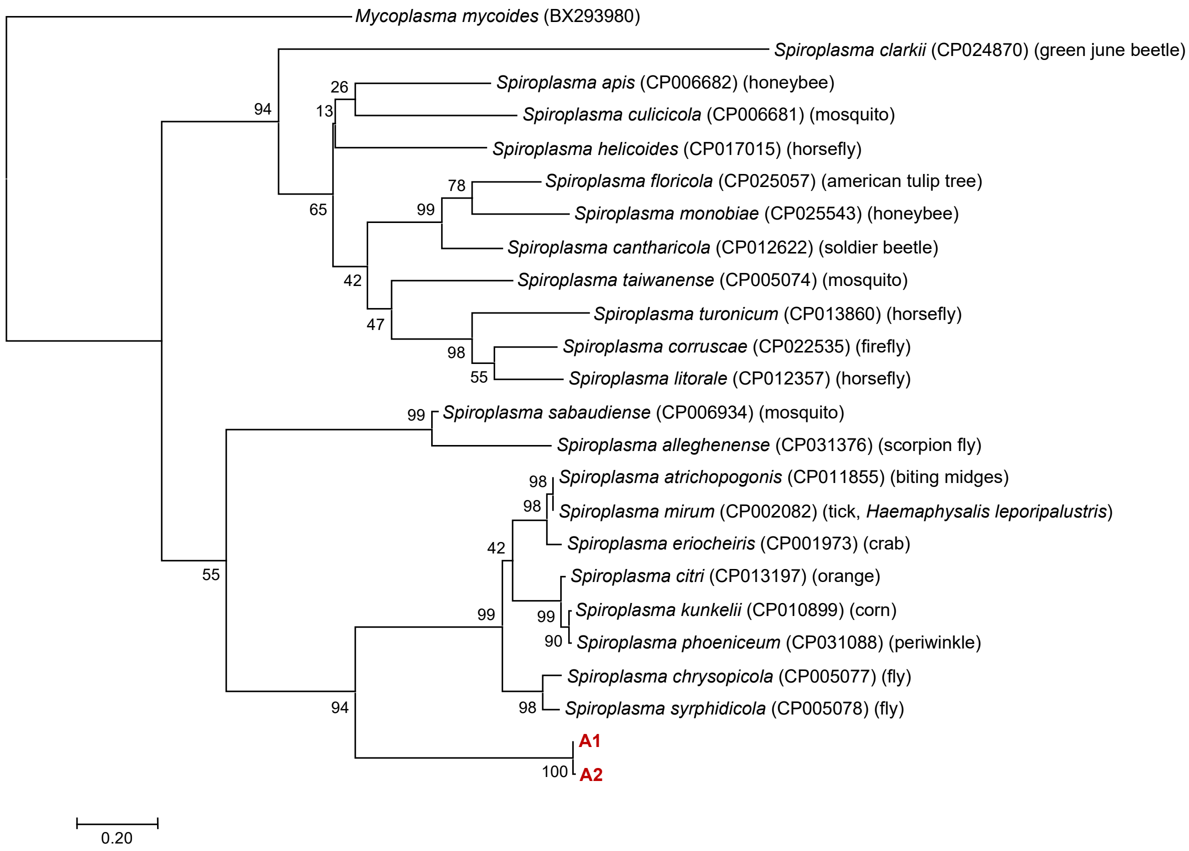

Figure S4. A phylogenetic tree based on the sequences of *dnaA* gene. The analysis was performed using a maximum-likelihood method based on the General Time Reversible model. A discrete Gamma distribution was used to model evolutionary rate differences among sites [5 categories (+G, parameter = 1.3300)] with bootstrap tests of 1,000 replicates in MEGA7. The sequences obtained in this study are included with allele names provided in Table 3 and are shown in red. The sequences of other *Spiroplasma* species were retrieved from GenBank.
